# Chemical Characterization and Biological Activity of the Essential Oil from *Araucaria brasiliensis* Collected in Ecuador

**DOI:** 10.3390/molecules27123793

**Published:** 2022-06-13

**Authors:** Diana Jaramillo, James Calva, Nicole Bec, Christian Larroque, Giovanni Vidari, Chabaco Armijos

**Affiliations:** 1 Departamento de Química, Universidad Técnica Particular de Loja, Loja 1101608, Ecuador; dsjaramillo2@utpl.edu.ec (D.J.); jwcalva@utpl.edu.ec (J.C.); 2 Institute for Regenerative Medicine and Biotherapy (IRMB), Université de Montpellier, INSERM, 34295 Montpellier, France; nicole.bec@inserm.fr (N.B.); cjlarroque@gmail.com (C.L.); 3 Medical Analysis Department, Faculty of Applied Science, Tishk International University, Erbil 44001, Iraq; vidari@unipv.it

**Keywords:** *Araucaria brasiliensis*, essential oil, chemical characterization, enantioselective analysis, AChE, BuChE

## Abstract

The purpose of this study was to determine the chemical composition, physical properties, enantiomeric composition and cholinesterase inhibitory activity of the essential oil (EO) steam-distilled from the leaves of the plant *Araucaria brasiliensis* Loud. collected in Ecuador. The chemical composition was determined by gas chromatography coupled to mass spectrometry (GC-MS) analysis on two capillary GC columns (DB5-ms and HP-INNOWax). Thirty-three compounds were identified in the EO; the main compounds were beyerene (26.08%), kaurene (24.86%), myrcene (11.02%), α-pinene (9.99%) and 5,15-rosadiene (5.87%). Diterpene hydrocarbons (65.41%), followed by monoterpene hydrocarbons (21.11%), were the most representative components of the EO. Enantioselective analysis of the EO showed four pairs of enantiomeric compounds, α-pinene, camphene, γ-muurolene and δ-cadinene. In an in vitro assay, the EO showed moderate inhibitory activity towards the enzyme butyrylcholinesterase (BuChE) (95.7 µg/mL), while it was inactive towards acetylcholinesterase (AChE) (225.3 µg/mL). Further in vivo studies are needed to confirm the anticholinesterase potential of the EO.

## 1. Introduction

The 19 species belonging to the genus *Araucaria* (family Araucariaceae) [1] are evergreen coniferous trees, several of which are used for ornamental and timber purposes worldwide [2]. Two species grow in Latin America (Chile, Argentina and Brazil), whereas the others are distributed in Southwest Pacific regions from Australia to New Caledonia, Papua New Guinea and the Norfolk Islands [3,4]. *Araucaria* plants exhibit different pharmacological activities, such as anti-inflammatory [5], antiviral, neuroprotective, anti-depressant, anti-coagulant [2], antifungal and antibacterial effects [6]. Phytochemicals of various types were isolated from *Araucaria* taxa, including flavonoids, sesquiterpenes, diterpenes and phenylpropanoids [7,8,9]; in addition, the composition of the essential oils (EOs) of some species was analyzed, such as EOs from *A. hunsteinii* K. Schum., *A. luxurians* (Brongn. & Gris) de Laub., *A. montana* Brongn. & Gris, *A. muelleri* (Carrière) Brong. & Gris., *A. scopulorum* de Laub. [10], *A. columnaris* (G. Forts) [11] and *A. angustifolia* (Bertol.) Kuntze [12].

The tree *Araucaria brasiliensis* Loud. (a synonym of *A. angustifolia* (Bertol.) Kuntze) grows in the wild in the forests of southern Brazil, Argentina, Paraguay and Chile; it is one of the most important natural biomes [13], and it is the most economically important conifer native to Brazil, where the leaves are used as an emollient, an antiseptic and to treat respiratory infections and rheumatism [12]. In Ecuador, due to its large size and its spectacular branching, it is used in the decoration of parks, avenues and botanical gardens, as well as for timber.

Two major lectins (lectin I and lectin 11) were isolated from the seeds of *A. brasiliensis* [14], which, in Brazil, is called “pinhão”. They are consumed by the local people as a traditional high-calorific foods [15] in winter and are eaten in the wild by birds and rodents. Flavonoids were identified in the leaves of *A. brasiliensis*, including six main biflavones, amentoflavone, mono-, di-, tri and tetra-*O*-methylamentoflavone and ginkgetin [16]. The last compound exhibits anti-HSV-1, antineoplastic and inhibitory activities towards arachidonate 5-lipoxygenase and cyclooxygenase 2.

We were interested in exploring whether the phytochemicals of *A. brasiliensis* could justify its inclusion in the *Araucariaceae* family. In this paper, we report for the first time the composition of the EO from the leaves collected in Ecuador, which were compared with those of other members of the *Araucariaceae* family.

No current protocol can be proposed to cure patients suffering from neurological disorders such as Alzheimer’s disease (AD), senile dementia, ataxia and myasthenia gravis; however, some medications may help to change the disease progression or mitigate some of the symptoms. Acetylcholinesterase (AChE) and/or butyrylcholinesterase (BuChE) inhibitors, such as donepezil, rivastigmine or galantamine, are the only drugs recognized for Alzheimer’s disease (AD) treatment. They prevent the breakdown of the neuromediator acetylcholine, which is essential for synaptic communication, and are possibly associated with a glutamate regulator. However, due to their modest efficacy and adverse effects, the use of these drugs is controversial. Therefore, there is a constant need for new treatments to treat or lower cognitive, Alzheimer-related symptoms, and the inhibition of acetylcholine breakdown remains the favorite route. In a normal brain, AChE represents 80% of the activity and BuChE the remaining 20%. In an Alzheimer’s-disease (AD) brain, BuChE activity rises, while AChE activity remains unchanged or declines. It has been demonstrated that selective inhibition of BuChE not only increases the acetylcholine level significantly but also improves memory in elderly rats. In this context, natural products and extracts have shown therapeutic potential to help in slowing down the evolution of this devastating disease [17,18]. On the other hand, essential oils (EOs) showing cholinesterase inhibitory activity are considered potential neuroprotective remedies for age-related neurodegenerative diseases [19,20,21], and the EOs of different plants growing in Southern Ecuador have shown moderate cholinesterase activity [22,23,24,25,26]. Specifically, the oils exhibited interesting, selective BuChE inhibitory activity. In continuation of these studies, we tested the still unknown inhibitory activity of the leaf EO from *A. brasiliensis* against the enzymes AChE and BuChE.

## 2. Results

### 2.1. Physical Properties of the EO

Steam distillation of *A. brasiliensis* leaves gave an odorous yellow EO with a yield of 0.042% ± 0.022 (*v*/*w*) from fresh plant material. The physical properties at 20 °C, as the mean of three analyses, were: relative density = 0.799 ± 0.035; refractive index = 1.511 ± 0.004; optical rotation = −3.920 ± 0.002.

### 2.2. Chemical Composition of the EO

**Figure 1:**
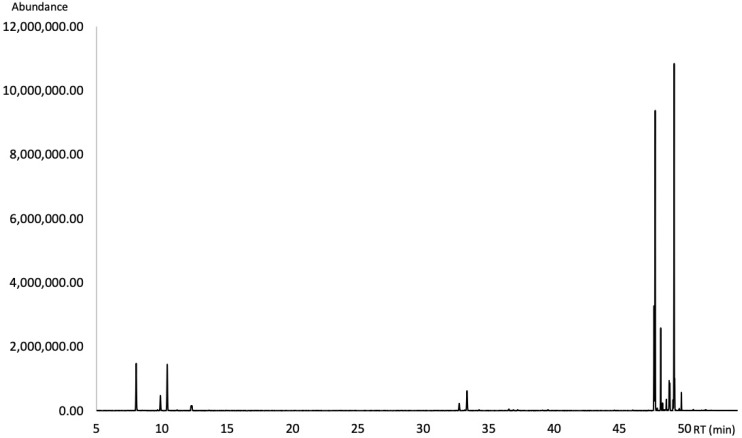
Gas chromatogram of the leaf essential oil of *A. brasiliensis* on a DB-5ms column.

**Figure 2:**
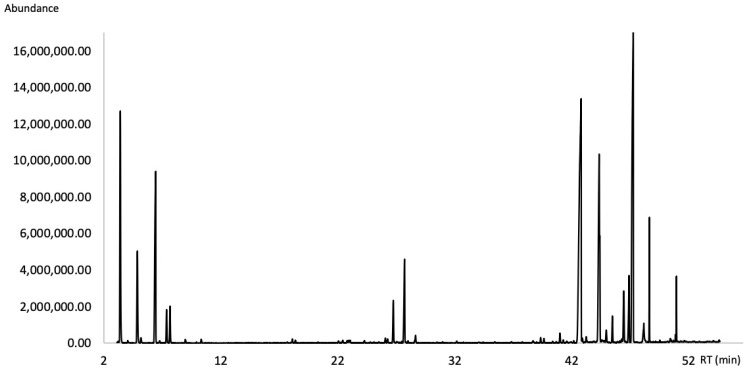
Gas chromatogram of the leaf essential oil of *A. brasiliensis* on a HP-INNOWax column.

**Figure 3:**
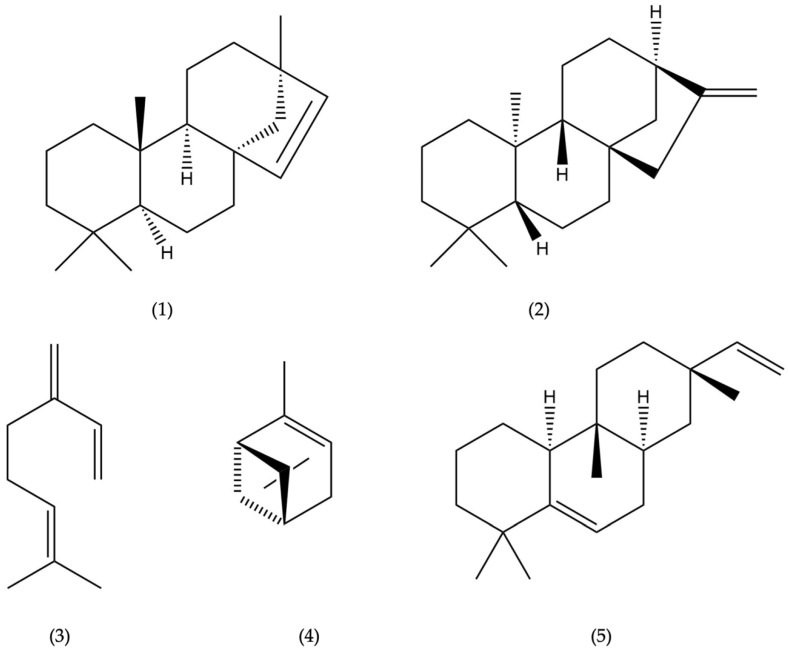
Chemical structures of the main compounds of the leaf essential oil of *A. brasiliensis*: beyerene (**1**), kaurene (**2**), myrcene (**3**), α-pinene (**4**) and rosa-5,15-diene (**5**).

Thirty-three compounds were identified in the EO by using GC-MS and GC-FID techniques, accounting for 88.16% of the EO analyzed on a non-polar DB-5ms capillary column (Figure 1) and 99.07% of the EO analyzed on a polar HP-INNOWax capillary column (Figure 2). The DB-5ms analysis showed that the EO consisted mainly of diterpene hydrocarbons (65.41%), followed by monoterpene hydrocarbons (12.55%) and sesquiterpene hydrocarbons (6.10%). Oxygenated terpenoids occurred in the oil as minor components. Qualitative and quantitative analytical results are summarized in Table 1. The chemical structures of the major compounds of the EO are shown in Figure 3.

### 2.3. Enantiomeric Analysis of the EO

The enantioselective analysis of the EO from *A. brasiliensis* leaves was performed, for the first time, on a cyclodextrin-based chiral column. Four pairs of enantiomers were detected, which were accurately quantified. The linear retention indices, enantiomeric distribution and percent enantiomeric excess (ee%) of each pair are shown in Table 2. The elution order of the enantiomers was determined by the injection of enantiomerically pure standards.

### 2.4. Cholinesterase Inhibition Assay of the EO

**Figure 4:**
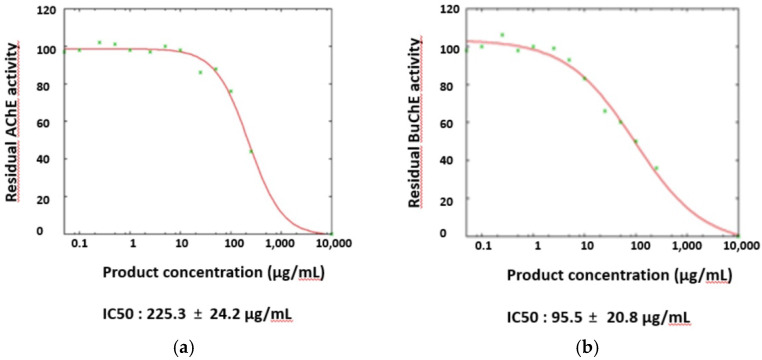
Calculation of the IC_50_ values of *Araucaria brasiliensis* EO towards (**a**) acetylcholinesterase (AChE) and (**b**) butyrylcholinesterase (BuChE).

The anti-cholinesterase activity of the EO from *A. brasiliensis* was evaluated by a colorimetric method (see the experimental section). The EO showed weak inhibitory activity towards the enzyme AChE and moderate inhibitory effects towards BuChE. Donepezil hydrochloride was used as a positive control (Table 3, Figure 4).

## 3. Discussion

The yield of the EO steam-distilled from *A. brasiliensis* leaves collected in Ecuador was rather low (0.042% (*v*/*w*)), in accordance with the yields of the EOs isolated from *A. cunnighamii* (=0.09%) and *A. heterophylla* (=0.10%) from India [39], *A. heterophylla* (=0.25%) and *A. bidwillii* (=0.05%) from Egypt [40] and *A. heterophylla* (=0.20%) from Hawaii [41]. However, the yield was slightly higher than the yield (=0.03%) reported for the EO isolated from the leaves of *A. angustifolia* collected at the Royal Botanic Gardens, Sydney, Australia [10]. Moreover, due to the large amount of biomass produced by a tree, scale up of the oil extraction might be interesting. Possible seasonal variation in the EO yield should also be examined to establish the harvesting time of the plant with the highest oil yield [42].

The most abundant chemical components identified in the leaf EO of *A. brasiliensis* from Ecuador were beyerene (1) (26.08%), kaurene (2) (24.86%), myrcene (3) (11.02%), α-pinene (4) (9.99%) and rosa-5,15-diene (5) (5.87%) (Figure 3). This finding is in contrast with the content of the EO from the leaves of *A. angustifolia* collected in Australia that contained sesquiterpene germacrene-D (8.6%) and diterpenes hibaene (29.7%) and phyllocladene (20.1%) as the main components [10]. On the other hand, Verma and collaborators determined beyerene (34.6–44.4%), caryophyllene oxide (0.5–17.9%), α-pinene (3.3–16.2%), germacrene D (0.1–9.8%), kaurene (1.7–5.1%) and 13-*epi*-dolabradiene (4.2–4.8%) as the main constituents of the EO from the foliage of *A. cunninghamii* from India [39], whereas the EO of *A. heterophylla* from India was dominated by diterpene hydrocarbons, among which 13-*epi*-dolabradiene (42.7%) and beyerene (22.2%) were the main constituents [39]. On the other hand, α-pinene was the main volatile component identified in the leaf oil of *A. heterophylla* from Egypt, accounting for 70.85%, followed by limonene (4.26%), phyllocladene (3.3%), ɣ-terpinene (3%), germacrene D (2.99%), β-caryophyllene (2.93%), sabinene (1.51%) and camphene (1.13%) [40], whereas the leaf oil from Australian *A. heterophylla* was dominated by the monoterpene α-pinene (52.4%) followed by the diterpene phyllocladene (32.2%) [10]. Instead, Hawaiian *A. heterophylla* foliage oil was characterized by β-caryophyllene (24.4%), while β-pinene was present only as traces (0.2%) [41]. The main constituents of *A. bidwillii* essential oil from Egypt were (+)-beyerene (35.65%), *trans*-nerolidol (13.66%), ɣ-elemene (6.09%), germacrene D (5.53%), τ-muurolol (2.51%), τ-cadinol (1.76%), α-pinene (1.52%) and kaur-15-ene (1.37%) [40], while, in the EO of Australian *A. bidwillii*, the content of mono- and sesquiterpenes was lower and higher than that of diterpenes, respectively, with beyerene being the main constituent (76%) [10]. In contrast, the leaf essential oil of *A. bidwillii* from Germany was mainly composed of monoterpene and sesquiterpene hydrocarbons with only a minor portion of diterpenes [43].

These data indicate the existence of interspecific and even intraspecific variation of *Araucaria* EOs, possibly due to the existence of genotypes. However, it is well known that the chemical composition of an EO, qualitatively and quantitatively, may also depend on environmental conditions, seasonality, altitude, geographical source of the plant, isolation procedure, etc. It should be noted that essential oils from *Araucaria* species, including the leaf EO of *A. brasiliensis* from Ecuador, are usually rich in diterpenes, and this characteristic rarely occurs in nature [39,40]. The tetracyclic diterpenes, beyeranes, occur only in a few species of Cupressaceae and Araucariaceae, whereas kauranes are widely distributed in the conifer families, especially in Araucariaceae [40].

The enantiomeric distribution and the percent enantiomeric excess (ee%) of the EO components of *A. brasiliensis* from Ecuador were determined by enantioselective GC-MS analysis on a cyclodextrin-based chiral stationary phase. Four pairs of enantiomers were identified, including two monoterpene and two sesquiterpene hydrocarbons (Table 2). The ee% = 98.94 of α-pinene was the highest among the four compounds, indicating that this monoterpene was almost enantiomerically pure. Instead, the enantiomeric excesses of the other three compounds were rather low. The combination of chemical and chiral features of an EO is very useful for assessing its identity and quality. Moreover, interactions with chiral entities, such as enzymes, proteins, and receptors, may vary greatly between enantiomers, causing significantly different biological activities such as those related to pharmacology, pharmacokinetics, metabolism, toxicity, or immune response [44].

The EO from *A. brasiliensis* contained about 10% α-pinene, which was reported to exhibit potent effects against AChE [20,44]. Contrary to our expectations, however, the oil was almost inactive against AChE, whereas it exhibited moderate activity against BuChE. This finding indicates that the relationship between terpenoid structures and cholinesterase activity is rather complex, so it would be interesting to determine the individual activity of other components of the oil from *A. brasiliensis*. However, EOs are complex mixtures of constituents, and it has been suggested that, usually, it is not a single compound that is responsible for the biological effects; it may depend on several components that act in a synergistic manner or on compounds which mutually regulate each other [24].

## 4. Materials and Methods

### 4.1. Plant Material

Three lots of *A. brasiliensis* Loud. fresh leaves were collected in October 2019 from three 30-year-old trees growing on the campus of the Universidad Técnica Particular de Loja (UTPL) in the city of Loja, Southern Ecuador, at 2400 m above sea level (3°59′12″ S, 79°11′56″ W). The plant was identified by Nixon Cumbicus, curator of the Herbarium of the Universidad Técnica Particular de Loja (HUTPL); a voucher specimen has been deposited in the UTPL herbarium (HUTPL) with the accession number HUTPL-079.

### 4.2. Isolation of the Essential Oil

The EO was obtained by three steam distillations, each 3 h long, of 1000, 980 and 1110 g, respectively, of fresh leaves, using a Clevenger-type apparatus. The three EO samples were brought together, dried over anhydrous Na_2_SO_4_, filtered and stored in brown vials at 4 °C until analysis [24].

### 4.3. Physical Properties of the Essential Oil

The relative density of the oil was determined at 20 °C according to the international standard method AFNOR NF-T75-111 (ISO 279: 1998). The refractive index was measured at 20 °C on an ABBE refractometer according to the AFNOR NF 75-112 (ISO 280:1998) international standard method. The specific rotation was determined on an automatic polarimeter (Hanon-P-810) according to the international standard ISO 592-1998 guidelines. Each test was performed in triplicate, and an average value was calculated [24].

### 4.4. Chemical Composition of the Essential Oil

The chemical characterization of the leaf EO of *A. brasiliensis* was carried out both qualitatively and quantitatively. The qualitative analysis was performed by GC-MS on an Agilent 6890N Gas Chromatograph coupled to an Agilent 5973 Mass Selective Detector operating in electron-ionization mode at 70 eV. Two types of chromatographic column were used, a non-polar capillary column (DB5-ms, 5% phenyl-methylpolysiloxane stationary phase; 30 m × 0.25 mm i.d. × 0.25 μm of film thickness) and a polar capillary column (HP-INNOWax, polyethylene glycol stationary phase; 30 m × 0.25 mm i.d. × 0.25 μm of film thickness); helium was used as the carrier gas in constant flow mode (1.00 mL/min). The GC oven operated with a temperature ramp from 60 °C to 250 °C with a gradient of 3 °C/min; the ion source temperature was set at 250 °C. The EO was dissolved in dichloromethane, and 1 μL was injected in each analysis.

The quantitative analysis was conducted on an Agilent Gas Chromatograph (6890 series) with a flame ionization detector (CG-FID) using the two capillary chromatographic columns cited above. The other chromatographic conditions were the same as in the GC-MS analysis. The percentage of each identified component of the oil was calculated from the area of the corresponding CG-FID peak with respect to the total area of peaks without applying any correction factor. Average values and standard deviations were calculated from the results of the three injections. EO samples were prepared and analyzed under the same conditions as the GC-MS analysis [22,26].

### 4.5. Identification of the EO Components

The EO chemical components were identified by comparing their calculated linear retention indices (LRIs) and the EI-MS spectra with those of compounds with close retention indices reported in the literature. Comparison of the indices was considered reasonable in a range of ±20 units. LRIs were determined according to Van Den Dool and Kratz [45] relative to the retention times of a series of homologous *n*-alkanes from C_9_ to C_24_ (C_9_ from BDH, purity 99%, and C_10_–C_24_ from Fluka, purity 99%), which were injected on the DB5-MS and HP-INNOWax columns immediately after the EO under identical chromatographic conditions.

### 4.6. Enantioselective Analysis

The enantioselective analysis of the EO was performed by injecting samples of the EO into the same GC-MS system used for the qualitative analysis equipped with a capillary column with a 2,3-diethyl-6-*tert*-butyldimethylsilyl--cyclodextrin stationary phase. The chromatographic conditions were the same as for the GC-MS analysis. The homologous series of *n*-alkanes was also injected to calculate the linear retention indices of the enantiomers [26].

### 4.7. Cholinesterase (ChE) Inhibition Assay

The cholinesterase (ChE) inhibitory activities of the EO from *A. brasiliensis* leaves against AChE (from *Electrophorus electricus*, Sigma-Aldrich, C3389, St Louis, MO, USA) and BuChE (from equine serum, Sigma-Aldrich, SRE020, St Louis, MO, USA) were measured by a colorimetric procedure adapted from Ellman et al. [46]. A typical 200 mL inhibition assay volume contained a phosphate-buffered saline solution (pH 7.4), DTNB (1.5 mM), and the tested sample dissolved in DMSO (final concentration = 1% *v*/*v*). Both AChE (Type V-S, lyophilized powder, 744 U/mg solid, 1272 U/mg protein) and BuChE (lyophilized powder, 900 U/mg protein) were dissolved in PBS pH 7.4 and were used at 25 mU/mL for the assay. After 10 min of pre-incubation, the substrate, acetylthiocholine iodide (1.5 mM), was added to start the reaction. After 1 h incubation at 30 °C, 96-well microtiter multiplates were read by a Pherastart FS (BMG Labtech) detection system. Enzymatic activities were tested in the presence of the EO (from 0.05 to 250 mg/mL, see Figure 4) dissolved in DMSO, the concentration of which was kept constant. Donepezil was used as a reference inhibitor for both enzymes. The results were expressed as the mean ± SD of the three replicates. IC_50_ values were determined from a nonlinear regression model using the online GNUPLOT package (www.ic50.tk, www.gnuplot.info, accessed on 30 April 2022). Possible false positives due to high concentrations (>100 mg/mL) of amino or aldehyde compounds were not excluded [46].

## 5. Conclusions

The EO from the leaves of *A. brasiliensis* collected in Southern Ecuador was analyzed for the first time by GC-MS. It contained a high number of terpene hydrocarbons. The most abundant components were diterpene (65.41%), followed by monoterpene (25.55%) and sesquiterpene hydrocarbons (4.84%). The most abundant identified compounds were beyerene (1) (26.08%), followed by kaurene (2) (24.86%), myrcene (3) (11.02%), α-pinene (4) (9.99%) and rosa-5,15-diene (5) (5.87%). This composition was significantly different from that of the EOs from the other *Araucaria* taxa examined so far, notably from the composition of the EO isolated from *A. angustifolia* (synonym *A. brasiliensis*) collected in Australia [10]. This finding indicates the possible existence of genotypes and may be useful for assessing the identity and quality of the leaf oil of Ecuadorian *A. brasiliensis*. The enantioselective analysis of the oil, performed for the first time, indicated the presence of four non-racemic pairs of enantiomers. Moreover, the EO of *A. brasiliensis* exhibited an interesting, selective inhibitory activity against the enzyme BuChE with a value of IC_50_ = 95.7 µg/mL, indicating potential neuroprotective effects, for example, against Alzheimer’s disease (AD). However, further in vivo studies are needed to confirm the anticholinesterase potential of the EO.

## Figures and Tables

**Table 1:** Chemical composition of *Araucaria brasiliensis* leaf essential oil (EO).

		DB5-ms				HP-INNOWax			
N°	Compound	LRI ^a^	LRI ^b^	*X* ± SD	Ref.	LRI ^a^	LRI ^b^	*X* ± SD	Ref.
1	α-Pinene	931	932	9.99 ± 2.81	[27]	1062	1066	6.32 ± 1.01	[22]
2	Camphene	948	946	0.08 ± 0.02	[27]	-	-	-	
3	Sabinene	971	969	0.12 ± 0.04	[27]	1122	1121	0.20 ± 0.05	[23]
4	β-Pinene	976	974	3.34 ± 0.73	[27]	1111	1112	4.68 ± 1.02	[23]
5	Myrcene	989	988	11.02 ± 1.97	[27]	1168	1170	13.41 ± 0.98	[28]
6	α-Phellandrene	1006	1002	0.16 ± 0.05	[27]	-	-	-	
7	α-Terpinene	1016	1014	0.07 ± 0.02	[27]	1176	1185	0.06 ± 0.01	[29]
8	β-Phellandrene	1030	1025	1.90 ± 0.39	[27]	1209	1210	1.25 ± 0.27	[30]
9	γ-Terpinene	1057	1054	0.08 ± 0.01	[27]	1244	1245	0.11 ± 0.02	[31]
10	Terpinolene	1084	1086	0.07 ± 0.02	[27]	1281	1280	0.11 ± 0.03	[30]
11	Aromadendrene	1374	1374	0.07 ± 0.03	[27]	1594	1604	0.13 ± 0.03	[32]
12	Guaia-6,9-diene	1436	1439	0.07 ± 0.03	[27]	1597	1594	0.15 ± 0.03	[33]
13	γ-Muurolene	1440	1442	0.07 ± 0.02	[27]	1679	1676	0.18 ± 0.04	[34]
14	Germacrene D	1473	1478	1.11 ± 0.13	[27]	1698	1700	1.51 ± 0.12	[30]
15	Viridiflorene	1479	1480	0.11 ± 0.05	[27]	1685	1679	0.14 ± 0.03	[35]
16	Bicyclogermacrene	1489	1496	3.14 ± 0.21	[27]	1725	1722	4.08 ± 0.38	[36]
17	δ-Cadinene	1493	1500	0.15 ± 0.02	[27]	1750	1750	0.29 ± 0.04	[30]
18	β-Copaen-4-α-ol	1511	1513	0.18 ± 0.16	[27]	-	-	-	
19	Viridiflorol	1516	1522	0.19 ± 0.04	[27]	2075	2069	0.16 ± 0.01	[37]
20	Globulol	1556	1559	0.14 ± 0.04	[27]	2066	2057	0.27 ± 0.09	[37]
21	α-Muurolol	1560	1575	0.12 ± 0.04	[27]	-	-	-	
22	*epi*-α-Cadinol	1567	1567	0.15 ± 0.02	[27]			-	
23	Octadec-1-ene	1574	1590	0.10 ± 0.03	[27]	-	-	-	
24	Rosa-5,15-diene (Rimuene)	1592	1592	5.87 ± 1.01	[27]	2240	2255	8.00 ± 0.24	[38]
25	Beyerene	1642	1644	26.08 ± 3.65	[27]	2186	-	25.78 ± 1.55	
26	Pimaradiene	1653	1638	0.24 ± 0.04	[27]	-	-	-	
27	Sandaracopimara-8(14),15-diene	1795	1789	4.47 ± 0.84	[27]	-	-	-	
28	Sclarene	1902	1900	0.54 ± 0.15	[27]	2262	-	0.52 ± 0.04	
29	Kaur-15-ene	1933	1933	0.60 ± 0.11	[27]	2280	-	0.76 ± 0.03	
30	13-*epi*-Manool oxide	1939	1931	2.31 ± 1.07	[27]	2360	2376	2.17 ± 0.16	[32]
31	Kaurene	1950	1948	24.86 ± 2.21	[27]	2386	2399	29.24 ± 0.97	[34]
32	Abietadiene	1970	1968	0.14 ± 0.02	[27]	2450	2450	0.14 ± 0.01	[32]
33	Phyllocladanol	2000	1997	0.22 ± 0.01	[27]	-	-	-	
Monoterpene hydrocarbons (%)				12.55				21.05	
Sesquiterpene hydrocarbons (%)				6.10				6.72	
Oxygenated sesquiterpenes (%)				0.93				0.87	
Diterpenes hydrocarbons (%)				65.41				68.16	
Oxygenated diterpenes (%)				3.12				2.17	
Others (%)				0.05				0.10	
Total (%)				88.16				99.07	

LRI ^a^ = calculated linear retention index; LRI ^b^ = linear retention index from reference; Ref. = reference; *X* ± SD = percentage in the oil and standard deviation; both values were calculated as the means of three determinations; (-) unidentified compound on HP-INNOWax column.

**Table 2:** Enantioselective analysis of constituents of the leaf EO of *Araucaria brasiliensis*.

Component	RT ^a^ (min)	LRI ^b^	Enantiomeric Distribution (%)	ee (%)
(+)-α-Pinene	10.835	982	0.53	98.94
(-)-α-Pinene	10.935	984	99.47	
(+)-Camphene	11.830	999	43.97	12.86
(-)-Camphene	12.003	1000	56.03	
(-)-γ-Muurolene	45.013	1552	33.17	33.66
(+)-γ-Muurolene	45.111	1553	66.83	
(+)-δ-Cadinene	48.097	1607	38.51	22.98
(-)-δ-Cadinene	48.191	1609	61.49	

RT ^a^, retention time; LRI ^b^, calculated linear retention index; ee (%), percent enantiomeric excess.

**Table 3:** Cholinesterase inhibitory activity.

Sample	AChE, IC_50_ ± SD (µg/mL)	BuChE, IC_50_ ± SD (µg/mL)
*Araucaria brasiliensis EO*	225.3 ± 24.2	95.7 ± 20.8
*Donepezil hydrochloride*	0.04 ± 0.01	3.60 ± 0.20

## Data Availability

Not applicable.

## Sample Availability

Samples of the compounds are available from the authors.
